# Motor Competence in Individuals with Down Syndrome: Is an Improvement Still Possible in Adulthood?

**DOI:** 10.3390/ijerph19042157

**Published:** 2022-02-14

**Authors:** Federico Quinzi, Giuseppe Vannozzi, Valentina Camomilla, Maria Francesca Piacentini, Florin Boca, Eric Bortels, Eva Kathrein, Adrian Magyar, Fabio Verdone, Paola Sbriccoli

**Affiliations:** 1 Department of Human Movement and Health Science, University of Rome “Foro Italico”, 00135 Rome, Italy; fquinzi@libero.it (F.Q.); valentina.camomilla@uniroma4.it (V.C.); mariafrancesca.piacentini@uniroma4.it (M.F.P.); paola.sbriccoli@uniroma4.it (P.S.); 2 Romanian Karate Federation, 022103 Bucharest, Romania; bocazawa2@yahoo.com; 3 I-Karate Global Federation—IKANDO, 3500 Hasselt, Belgium; eric.bortels@i-k-f.net; 4 Karate Club Bregenz, 6900 Bregenz, Austria; evakathrein@karate-bregenz.at; 5 Hungarian Karate Federation, 1146 Budapest, Hungary; magyaradrianpal@gmail.com; 6 Italian Karate Federation—FIJLKAM, 00122 Rome, Italy; verdonefabio@outlook.com

**Keywords:** skill assessment, motor development, TGMD-3, karate, physical activity, adapted training

## Abstract

In children, motor competence (MC) and the amount of physical activity are tightly interconnected. In adults with Down syndrome (DS), MC has been poorly addressed, resulting in a limited understanding of the possibility to improve MC over time. Here, we aim to: (1) investigate MC in adults with DS by comparing them with a group of typically developed peers and (2) verify the effect of an adapted karate program on MC. Adults with DS (DSG; *n* = 57) and typically developed adults (TDG; *n* = 21) performed the Test of Gross Motor Development version 3 (TGMD-3). The total TGMD-3 score (^TOT^TGMD-3), the locomotor (^LOC^TGMD-3), and object control (^OBJ^TGMD-3) scores were computed. After a 40 week adapted karate program, DSG (*n* = 37) underwent the post-training TGMD-3 assessment. Compared to TDG, DSG showed lower ^TOT^TGMD-3 (DSG: 45.5 ± 17.3; TDG: 77.3 ± 9.5), ^LOC^TGMD-3 (DSG: 22.2 ± 10.0; TDG: 36.2 ± 7.6) and ^OBJ^TGMD-3 (DSG: 23.3 ± 10.9; TDG: 41.1 ± 5.6). After the training, ^TOT^TGMD-3, ^LOC^TGMD-3 and ^OBJ^TGMD-3 increased by 35.6%, 30.0% and 40.7%, respectively. Our results suggest that MC acquisition does not evolve into a mature form in adulthood in individuals with DS. Moreover, a brief exposure to an adapted karate program induces an increase in motor competence in DS, even in adulthood.

## 1. Introduction

Motor competence can be defined as the degree of proficiency in performing a variety of motor tasks, such as locomotor, object control, and balance, and the processes underlying these skilled performances [1,2]. Since the pivotal study by Seefeldt [3] in which the “proficiency barrier” concept was first introduced, a wealth of studies has flourished, focusing on motor competence [4,5]. This body of literature proposed the existence of a relationship between motor competence and the amount of daily physical activity in children [5,6,7,8,9]. Interestingly, this relationship was extended also to adults [10,11], showing that motor skill performance in ballistic skills correlates with several measures of physical fitness [10,11].

Individuals with intellectual disability risk being exposed to a low amount of physical activity in general and, specifically, to a low amount of sport-type physical activity. Therefore, it is not surprising that an impressive number of studies has been carried out on their motor competence. However, this body of knowledge focused mainly on children [12,13,14,15,16,17], paying particular attention to those with Down syndrome (DS) [18,19,20,21,22]. This research showed poorer general motor competence in children with DS compared to typically developed peers [18,21,22]. In particular, children with DS displayed poorer motor competence in both the locomotor and object control domains [18,22] and in every individual skill assessed with the Test of Gross Motor Development (TGMD) as compared to typically developed children. In children and adolescents with DS, previous literature hypothesized a delay in the acquisition of motor competence compared to typically developed peers [23,24]. However, due to the paucity of information on adults with DS, it cannot be established whether this population is eventually able to display a motor competence similar to that observed in typically developed adults and which skills, if any, remain affected by the syndrome in adulthood. In typically developed adults (TD), motor competence reaches a peak at about 19–25 years of age, followed by a slow decay until approximately 35 years, after which a steeper decrease in motor competence can be observed [25,26]. Unfortunately, similar research on adults with DS is not available. A couple of recent studies using a combined qualitative and quantitative approach [27,28] showed poorer motor competence in adults with DS compared to typically developed (TD) peers in both a locomotor and a ballistic skill. However, a comprehensive assessment of motor competence in adults with DS is still lacking. This information would establish whether the concept of a proficiency barrier [3] can be applied also in this population as a potential factor explaining the limited participation in physical activity and, consequently, to further our understanding of the mechanisms promoting an active lifestyle. Besides, an adequate assessment of motor competence through the observation of the fundamental movement skills of individuals with DS would allow the identification of developmental delays in specific domains in adult individuals with DS. This knowledge could help professionals to design specific physical activity programs to increase the overall motor competence by working in the aforementioned domains.

In addition to the above mentioned factors observed in individuals with Down syndrome, it could not be excluded that a possible poor motor competence observed in this population may also be accounted for by a lower exposure to physical activity programs compared to TD peers [29,30] and consequent delays in the acquisition of motor competence. Previous research has verified this possibility by including participants with DS in physical activity programs aiming at increasing strength and balance [13,31,32,33] or gross [12,34,35,36] and fine [37,38] motor skills. This body of research showed moderate to strong evidence of the beneficial effects of physical activity on strength and balance in adults with DS [32,33,39], while improvements in motor competence were shown only in children [34,35,40]. In particular, if collapsed together, these latter studies showed an average improvement in motor competence of approximately 30% after participation in physical activity programs, with the largest increase in their motor competence observed in the interventions specifically designed to improve locomotor and object control skills [40]. So far, no study directly investigated the effects of an adapted physical activity program on motor competence in adults with DS. To this end, an adult population seems particularly suitable to study chronic exposure to physical activity programs, in that adults would enable us to rule out possible maturation effects that could result in biased outcome measures.

Among the various interventions to increase motor competence in individuals with DS, martial arts, and especially karate, seem to benefit various domains of both physical and cognitive fitness, including flexibility, muscle strength, balance and coordination, as well as cognitive functions. Indeed, karate technical actions often rely on complex ballistic skills, which may foster increased intra- and intermuscular coordination [41,42,43]. Previous studies showed that the practice of karate is associated with increased upper [44] and lower limb strength [43] in adults and coordination skills [36] in children. Moreover, karate practice has been associated with increased postural control [45,46]. All these aspects argue for karate as a promising intervention strategy to increase motor coordination and motor competence.

Therefore, the aim of the present study is twofold: (i) to investigate motor competence in adults with DS and compare them with a group of TD adults and (ii) to verify the effect of an adapted karate physical activity program on motor competence in adults with DS. In regard to the first aim, in agreement with the previous literature on motor competence in children with DS, we expect a generally poorer motor competence in adults with DS compared to TD adults. As previously observed, we hypothesize a lower competence in hopping and kicking and, in general, we expect that the skills requiring a higher coordinative ability, such as those skills involving contralateral movements of the upper and lower limbs, shall be more impaired in DS compared to TD adults. In regard to the second aim, evidence on the beneficial effects of karate on strength and coordinative skills suggests that improvements in motor competence after participation in the physical activity program shall be expected. Due to the absence of studies on adults with DS, hypotheses on the magnitude of the improvements, if any, cannot be easily formulated. However, we expect greater improvements in the skills relying on strength (i.e., horizontal jump and sliding) rather than those relying on coordinative skills.

## 2. Materials and Methods—Study 1

### 2.1. Participants

Fifty-seven adult individuals diagnosed with Down syndrome (DSG) and twenty-one typically developed adults, serving as a control group (TDG), volunteered to participate in this study. Participants were recruited through local associations for individuals with DS and local karate associations. Participants were included in the study if they: (1) had no history of musculoskeletal injuries or major head concussions in the twelve months preceding the study and (2) had no contraindication to the practice of sports activity at a recreational level. The anthropometric characteristics of the two groups are reported in Table 1. After a full explanation of the procedures and aims of the study, which were approved by the local ethical committee, written informed consent was signed either by the participants or by their legal guardians, when appropriate.

### 2.2. Procedures

The participants’ motor competence was evaluated in a single session. For both groups, the evaluation of motor competence was carried out using the Test for Gross Motor Development version 3 (TGMD-3, [47]). This test encompasses thirteen skills belonging either to the locomotor (running, galloping, hopping, sliding, skipping, and horizontal jumping) or object control (two-hand striking, one-hand striking, overarm throwing, underarm rolling, stationary dribbling, kicking, and catching) classes. For each of these skills, depending on the skill, the TGMD-3 foresees three to five performance criteria. For each performance criterion, one point is awarded only if the skill execution fulfills that criterion. For each skill, the total score is the sum of the skill-related performance criteria (running: RU, galloping: GA, hopping: HO, sliding: SL, skipping: SK, horizontal jumping: HJ two-hand striking: TH, one-hand striking: OH, overarm throwing: OT, underarm rolling: UR, stationary dribbling: SD, kicking: KI, catching: CA). The total locomotor score (^LOC^TGMD-3), the total object control score (^OBJ^TGMD-3), and the total TGMD-3 score (^TOT^TGMD-3) are also defined as the sum of the relevant skill-related scores. After a full explanation of each skill, participants were allowed to perform two practice trials to familiarize themselves with the tasks. For each skill, participants then performed two valid attempts that were video recorded (Samsung A51, full-HD 1920 × 1080 resolution; 30 fps) and stored on a personal computer to be analyzed offline. Between the two attempts participants were granted two-minutes of rest. One trained scorer carried out the analysis in the pre- and post-intervention assessment. The scorer was aware of whether a given trial was a pre- or post-intervention and, in the case of post-intervention, the scorer did not have access to the pre-intervention score. The scorer was independent from the colleague who created the methodological approach proposed in the adapted karate training.

### 2.3. Physical Activity Level

Before commencing the motor competence assessment, participants or their legal guardians were asked to fill in the International Physical Activity Questionnaire–short form (IPAQ [48]). In agreement with the instructions of this questionnaire, participants were classified as inactive if their total IPAQ score fell below 700 Mets, sufficiently active if the total score ranged between 700 and 2519 Mets, and very active if their IPAQ score exceeded 2519 Mets.

### 2.4. Statistical Analysis

All the analyses detailed in the following paragraph were carried out using the Statistica software (v10; StatSoft Inc., Tulsa, OK, USA). For all analyses, statistical significance was set to α = 0.05.

The normal distribution of the variables of interest was verified through the Shapiro–Wilk test. The anthropometric characteristics and physical activity levels (IPAQ) of the two groups showed normal distributions, and between-group differences were tested via the independent samples *t*-test. Conversely, the TGMD-3 variables (^TOT^TGMD-3, ^LOC^TGMD-3, ^OBJ^TGMD-3, RU, GA, HO, SL, SK, HJ, TH, OH, OT, UR, SD, KI; and CA) did not show a normal distribution. Thus, between-group comparisons were carried out using separate Mann–Whitney U-tests. The standard error of the median (SEM) for the TGMD-3 variables were computed using the bootstrap method. Cohen’s *d* was used as a measure of the effect size, and it was computed from the Z distribution following the equation proposed by Fritz and colleagues [49]. The Bonferroni correction for multiple comparisons was adopted when appropriate.

## 3. Results—Study 1

**Figure 1:**
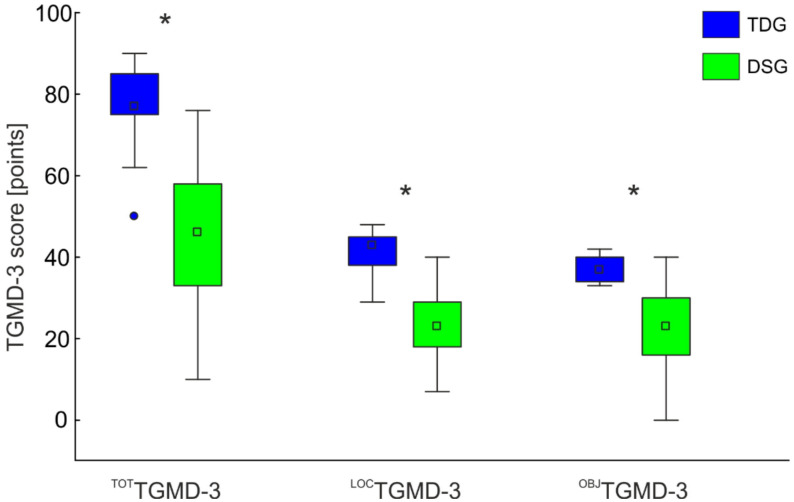
^TOT^TGMD-3, ^LOC^TGMD-3, ^OBJ^TGMD-3 scores for the DSG and TDG groups. The boxplots report the medians (squares in the box) and the 25%, and 75% limits. The whiskers report the data ranges. * Denotes significant differences between the two groups. The significance level was set to α = 0.05. The Bonferroni-corrected significance level was 0.017.

Figure 1 shows the average score of DSG and TDG in the Total (^TOT^TGMD-3), locomotor (^LOC^TGMD-3), and object control (^OBJ^TGMD-3) subtests. DSG showed lower scores than TDG in the ^TOT^TGMD-3 (Z_(76)_ = −6.15; *p* < 0.001; Cohen’s *d* = 1.99); ^LOC^TGMD-3 (Z_(76)_ = −5.36; *p* < 0.001; Cohen’s *d* = 1.55), and ^OBJ^TGMD-3 (Z _(76)_ = −6.19; *p* < 0.001; Cohen’s *d* = 2.01). 

When single skills were evaluated independently, DSG showed lower scores than TDG in all the skills, with the lowest score obtained in the skipping skill, followed by kicking and overarm throwing. Table 2 reports for both groups the scores for each skill and the relevant statistics.

The *t*-test performed on the results of the IPAQ showed that the participants of TDG were more active than those in the DSG (TDG: 4026.2 (3487.8); DSG: 1377.2 (1336.4); T_(76)_: −4.88, *p* < 0.001; Cohen’s *d* = 1.00). More specifically, this difference was mainly driven by the presence of higher rates of vigorous activity in TDG than in DSG (see Table 1). In agreement with the classification provided in the IPAQ, our TDG participants were classified as very active, whereas participants in the DSG were classified as sufficiently active.

## 4. Materials and Methods—Study 2

### 4.1. Participants

The same fifty-seven participants with DS that were included in study 1 were asked to participate in study 2, where the effects of an adapted karate training program, detailed in the following paragraph, on motor competence were evaluated. Only participants that were naïve to karate were considered eligible to participate in this study. Of the initial fifty-seven participants that underwent the initial evaluation in study 1, thirty-seven completed the entire training program and underwent the post-training assessment. This relatively high drop-out rate might be accounted for by the SARS-CoV-2 pandemic outbreak. Of the 20 participants who dropped out, 6 of them left immediately after the initial test and did not take part in the intervention, 12 took part in the intervention but were not able to participate in the post intervention assessment, and only 2 dropped during the training. The anthropometric characteristics of this group are reported in Table 3.

### 4.2. Procedures

This study was designed as an intervention study in which the motor competence of the DSG was evaluated before and after adapted karate training. Motor competence of the DSG enrolled in the adapted karate program was evaluated using the TGMD-3, and the data were processed as previously detailed in study 1.

### 4.3. Training

Participants with DS practiced adapted karate bi-weekly for one hour for forty weeks. The training program was carried out according to the methodology proposed by the I-Karate Global—IKFI (a detailed description of each training session is available at: https://www.ikons-project.eu/wp-content/uploads/2021/03/IKONS_IO2-3_ENG.pdf (accessed on 22 December 2021). Briefly, this training methodology encompasses coordinative exercises involving the upper and lower limbs of both sides of the body with the basic technical actions of karate i.e., punches, blocks, and kicks. The training is performed mostly in place and includes a few stepping actions, either forward or backward, on special mats depicting different colored shapes to guide participants in the action. These features are deemed to facilitate the practitioners to orient themselves in space.

### 4.4. Statistical Analysis

All the analyses detailed in the following paragraph were carried out using the Statistica software (v10; StatSoft Inc. Tulsa, OK, USA). For all analyses, statistical significance was set to α = 0.05.

The normal distribution of the variables of interest was verified through the Shapiro–Wilk test. The TGMD-3 variables (^TOT^TGMD-3, ^LOC^TGMD-3, ^OBJ^TGMD-3, RU, GA, HO, SL, SK, HJ, TH, OH, OT, UR, SD, KI, and CA) did not show a normal distribution and the effect of the adapted karate training program (PRE vs. POST) was evaluated using separate Wilcoxon rank-sum tests. Moreover, to verify whether the training-induced improvements in motor competence, if any, allowed DSG to reach at the end of the training a motor competence level comparable to that of the TDG for each specific skill tested, we compared the single skill scores obtained by the DSG in the post-training assessment (DSG-POST) with those of the typically developed group (TDG) through the Mann–Whitney U test. The standard error of the median (SEM) for the TGMD-3 variables was computed using the bootstrap method. Cohen’s *d* was used as a measure of the effect size, and it was computed from the Z distribution following the equation proposed by Fritz et al. [49]. The Bonferroni correction for multiple comparisons was adopted when appropriate.

## 5. Results—Study 2

**Figure 2:**
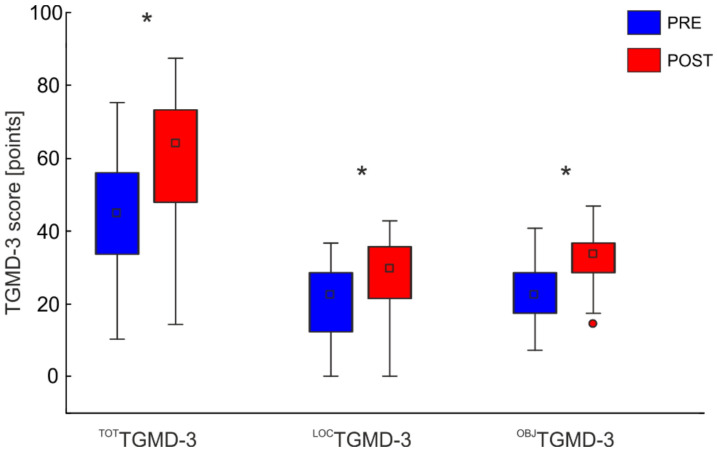
TGMD-3 total, locomotor, and object control scores before (blue bars) and after (red bars) the adapted karate physical intervention. The data are reported as means and SD. The significance level was set to α = 0.05. The Bonferroni-corrected significance level was 0.017. * Denotes significant differences between the pre and post intervention assessments.

The adapted karate intervention was well tolerated by the 37 participants who regularly attended the training sessions, with an average participation rate of 74%. Significant differences were observed between the pre- and post-intervention assessments for the ^TOT^TGMD-3 (Z_(36)_ = −5.08; *p* < 0.001) and for the two locomotor ^LOC^TGMD-3 (Z_(36)_ = −4.36; *p* < 0.001), and object control ^OBJ^TGMD-3 (Z_(36)_ = −4.86; *p* < 0.001) subtests, with the post-intervention assessment showing higher scores than the pre-intervention assessment (Figure 2).

The increase in the ^TOT^TGMD-3 score was associated with a different skewness of the distribution of the data between the pre- and post-intervention assessments. In the pre-intervention assessment, the ^TOT^TGMD-3 score followed a normal distribution, whereas in the post-intervention assessment it showed a shift in the distribution toward the right side of the distribution, with the largest number of observations occurring towards the higher scores (Figure 2, ^TOT^TGMD-3, and ^LOC^TGMD-3).

**Figure 3:**
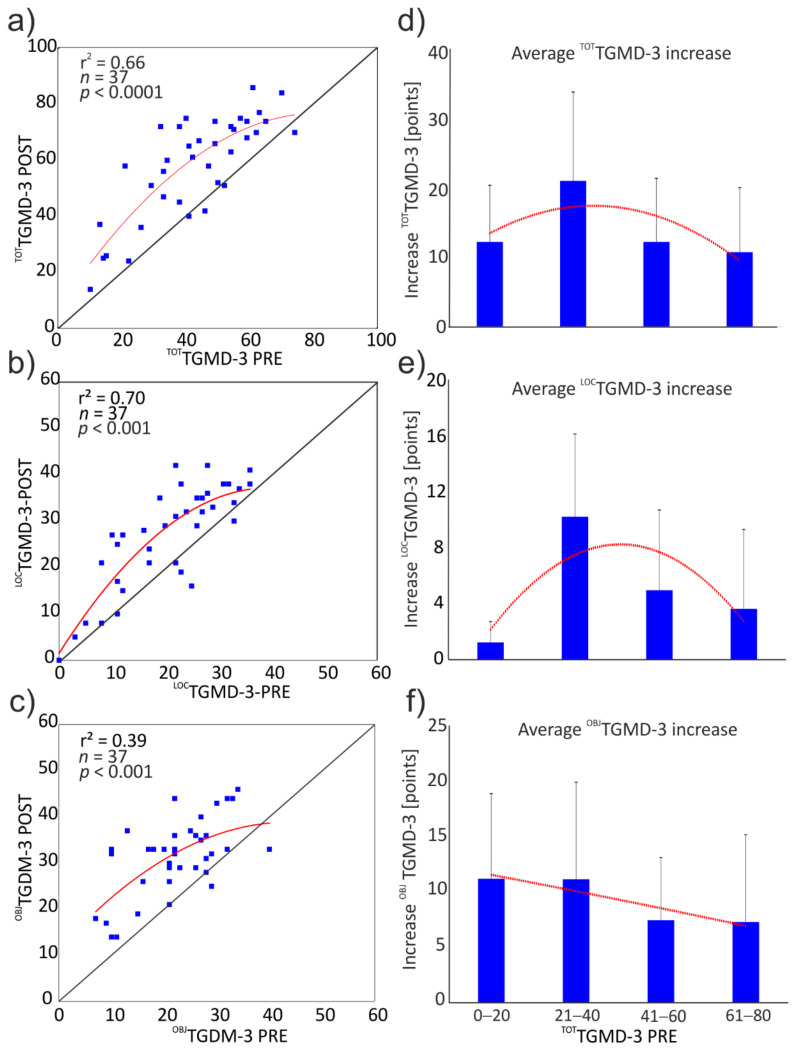
The left column proposes the scatterplots of the ^TOT^TGMD-3, ^LOC^TGMD-3, and ^OBJ^TGMD-3 scores measured in the pre- and post-intervention assessments, which are represented in the upper (**a**), middle (**b**), and lower panels (**c**), respectively. The identity line (straight black line) is indicated to highlight the variations in the scores; dots on the upper left side of the identity line indicate an increased score, whereas dots on the lower right side of the identity line indicate decreased score from pre- to post-intervention assessment. The data were fitted with a second-order polynomial (red line). The right column shows the average increase in ^TOT^TGMD-3 (**d**), ^LOC^TGMD-3 (**e**), and ^OBJ^TGMD-3 (**f**), grouped by pre-intervention ^TOT^TGMD-3 score.

The relationship between the ^TOT^TGMD-3 score of the pre- and post-intervention assessments is reported in Figure 3. All but four participants involved in the training increased their ^TOT^TGMD-3 score from pre- to post-intervention assessments (observations on the upper left side of the identity line in Figure 3a). The relationship between the pre- and post-intervention ^TOT^TGMD-3 score was modeled by fitting the data with a second-order polynomial line. This trend line showed that the participants with an average initial ^TOT^TGMD-3 score (range: 21–40) benefitted more from the proposed intervention. This holds also for the locomotor score, whereas, for the object control skills, those with the lowest initial ^TOT^TGMD-3 scores showed the largest changes.

When the single skills were considered independently, eight skills showed increased scores in the post- compared to the pre-intervention assessment (Table 4). Of these skills, running, horizontal jumping, and sliding belonged to the locomotor subtest (corresponding to 50% of the skills of the locomotor subtest), whereas one-hand striking, stationary dribbling, catching, kicking, and underhand roll belonged to the object control subtest (approximately 70% of the object control skills).

**Figure 4:**
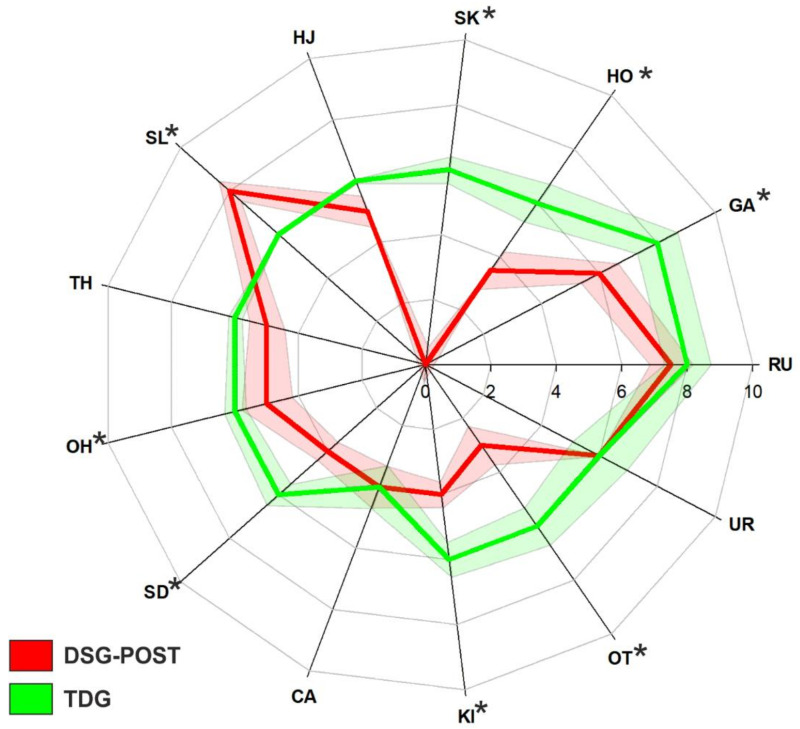
Radar plot showing the median score obtained by the DSG-POST (red line) and TDG (green line) in the single skills included in the TGMD-3. The red and green bands indicate the SEM for the two groups (same color code). The asterisks denote significant differences between the two groups at the 0.05 level.

After the adapted karate training intervention, the DSG group (DSG-POST) showed comparable motor competence to the TDG in five out of thirteen skills; motor competence was comparable between DSG-POST and TDG for running, catching, two-hand strike, and horizontal jumping (Figure 4).

## 6. Discussion

In agreement with our hypothesis, we showed that adults with DS present lower motor competence compared to their typically developed peers. Besides, the analysis performed at individual skill level allowed us to identify possible strength points of adults with DS. In the second part of this study, we investigated the effects of a physical activity course consisting of adapted karate on motor competence in adults with DS. In agreement with our hypothesis, adults with DS showed marked improvements in their overall motor competence, and, specifically, improved significantly in eight out of thirteen skills after the intervention.

Our results are in agreement with the previous literature investigating motor competence in children with DS [18,20,22]. Indeed, all these studies, using a previous version of the TGMD, showed lower motor proficiency in individuals with DS, both in locomotor and object control subtests compared to typically developing children. In particular, in agreement with Alesi et al. [18], who reported lower motor competence in children with DS for every skill tested, our adult participants with DS exhibited a lower motor competence than TD adults. Notably, in the locomotor subtests, the skill with the lowest score was the skipping skill, whereas in the object control subtest the lowest scores were observed for kicking and overarm throwing. Moreover, these results are consistent with previous observations of children with DS showing that skipping [18], kicking, and overarm throwing [20] were the skills mainly affected in this population. Indeed, skipping is a rhythmic action requiring, in addition to muscle power and dynamic balance, timely and accurate movements of bilateral body segments involved in the action. Similarly, overarm throwing requires, during the cocking phase, bilateral movement of upper and lower limbs in opposite directions. Previous studies argued that bilateral movements are particularly impaired in individuals with DS [50], possibly due to an impaired brain inter-hemispheric communication associated with a reduced volume of the corpus callosum and to the well-documented hypoplasia of the cerebellum, which is consistently involved in many bilateral coordinative tasks [51]. In regard to kicking, in a very recent study by our research group [28], we showed that the reduced motor competence in adults with DS is associated with a reduced angular velocity of the pelvis about the medio-lateral and cranio-caudal axes of the body, which we propose to be related to poor neuromuscular control of core muscles.

Concerning the second aim of the present study, we showed that, following an adapted karate intervention, improvements in motor competence occurred in adults with DS. This confirms that the poorer motor competence observed in the DSG can be ascribed, at least in part, to lower exposure to physical activity contexts. It is noteworthy that motor competence in eight out of thirteen skills was enhanced after the adapted karate program. Specifically, improvements in motor competence were ubiquitous for the locomotor and object control skills and were observed in running, horizontal jumping, sliding, one-hand striking, catching, kicking, and underhand roll. These increased proficiencies in the different motor skills were the result of improvements in the underlying mechanisms, such as strength, motor coordination, and balance. While the adopted ecological approach did not aim to selectively highlight the contribution of each component, notably, the literature already reports this information, as a result of karate training. Indeed, the practice of karate is associated with specific features of the neuromuscular system and, in more detail, with a preferential recruitment of fast twitch muscle fibers [42,43,44], favoring muscle force production. At the same time, karate practice is associated with enhanced intermuscular coordination, consisting of a fine-tuning of agonist and antagonist muscle activations [41,42,43] and with an improvement in general coordination [36]. Last, an improved in balancing ability has also been observed in karate practitioners [45,46]. Interestingly, significant positive correlations have been reported between motor competence in locomotor and object control skills and balance [20,50].

Our results on the effects of an adapted karate intervention are in line with previous research showing the significant effects of physical interventions on motor competence in individuals with DS [34,35,40]. It is worth noting that these studies proposed exercises encompassing skills similar to those included in the TGMD-3 [35,40]. Therefore, the improvements observed in these studies could be expected, possibly as a result of the learning effect. Only McGuire et al. [34], investigating the effect of an adapted dance program on motor competence, showed significant improvements in skills not directly performed in the training (walking, running, and jumping). However, this study was carried out on a small number of children with DS, thus limiting the possibility to generalize its results. Our results support implementing adapted training with this population in adulthood as well, aiming to improve motor competence. As proposed by previous literature [10], an improvement in motor competence might also induce an enrichment of the possibilities to be involved in physical activity or sport-type physical activity, possibly overcoming the conceptual proficiency barrier initially proposed by Seefeldt [3]. Indeed, a 40 week adapted karate intervention was sufficient to improve motor competence in the majority of the skills tested and reach the motor competence observed in the typically developed group in five out of thirteen screened skills (Figure 4).

Notably, the largest increases in motor competence, as assessed by the total and locomotor TGMD-3 score, were observed in those participants with a pre-training global motor competence falling approximately in the second quartile of the distribution (the range of 21–40 points in the ^TOT^TGMD-3). The reasons for such an improvement in this motor competence range are not easily identifiable, but some explanations, although purely speculative, could be proposed. It is conceivable that participants in this motor competence range may have benefitted from the training more than others, due to reduced exposure to physical activity prior to the adapted karate training compared to more motorically competent participants (in the third and fourth quartile) and, at the same time, their impairments due to the phenotypic features associated with the DS limit their motor competence to a lower extent compared to the participants in the first quartile.

Admittedly, the results of this study shall be considered in light of some limitations. The first limitation consists of the lack of a control group composed of adults with DS in study 2, not following a specific training program. This control group would have allowed us to precisely establish the effect of the adapted karate training, possibly ruling-out other confounding factors that could influence MC. Moreover, a control group would have enabled the exclusion of a potential, but not probable, learning effect on the skills proposed for the test. Although the possibility of a learning effect exists, some considerations advocate against this possibility. First, a previous version of the test (TGMD-2)s sharing 11 out of 13 skills with the TGMD-3, showed excellent psychometric properties with excellent test-retest reliability (ICC ranging from 0.81 to 0.98, [52]). The second consideration deals with the interval of time between the pre- and post-intervention assessments, which in the present study were spaced 10 months (40 weeks) apart. With such an an interval of time, it seems rather difficult that a learning process may occur in-between the two assessments, considering also that during the adapted karate program, trainers purposefully avoided including exercises other than those reported in the program (https://www.ikons-project.eu/wp-content/uploads/2021/03/IKONS_IO2-3_ENG.pdf) (Accessed on 22 December 2021). Relatedly, a second limitation of study 2 is the lack of a further control group involved in a generic physical activity program to assess the specificity of karate training in improving motor competence. Such a control group could have shed light on the specificity of the karate training program. Although this could provide crucial information, this was beyond the scope of the present study. Last, the assessment of possible improvements in cognitive functions following the adapted karate intervention would have allowed a comprehensive evaluation of the effect of an adapted karate program. Future studies are needed to investigate which physical intervention is more effective in improving motor competence and cognitive skills.

## 7. Conclusions

Overall, the present results showed that adults with DS present reduced motor competence compared to typically developed peers and suggest that, if no adjuvant actions are undertaken, adults with DS will not eventually be able to display a motor competence similar to their typically developed peers even in adult age. On the other hand, if compared to the age of our participants, the exposure to an adapted physical activity program has shown promising improvements in motor competence in adults with DS. Future studies could also verify if and how these improvements could be maintained over time, as a chronic positive effect of adapted training to improve motor competence in DS adults or to verify the effect of similar methodological approach in other populations with intellectual disabilities or developmental coordination deficits. Such an improvement may enrich the possibilities to be involved in physical and sports activities, thus promoting a healthy lifestyle and reducing medical costs. With this in mind, physical activity courses should be promoted in individuals with DS at adult ages as well. An adapted karate program may represent a valid and appealing possibility to increase physical activity levels, increase social inclusion, and, at the same time, improve motor competence.

## Figures and Tables

**Table 1:** Anthropometric characteristics of the two groups.

	DS (*n* = 57)	TDG (*n* = 21)	T-Value	*p*-Value
Age [years]	26.0 (7.4)	24.3 (2.1)	1.06	0.290
Mass [kg]	65.8 (14.5)	63.9 (11.6)	0.52	0.601
Height [m] *	1.54 (0.09)	1.66 (0.06)	−5.27	<0.001
BMI [kg/m^2^] *	27.5 (6.3)	23.5 (4.0)	2.79	0.006
IPAQ *	1377.2 (1336.4)	4026.2 (3487.8)	−4.88	<0.001
- Vigorous Activity *	155.7 (456.9)	2089.5 (2828.4)	−5.04	<0.001
- Moderate Activity	590.9 (848.5)	775.2 (584.1)	−0.91	0.360
- Walking	625.8 (501.5)	1159.3 (2025.4)	−1.85	0.067
Females/Males	21 (37%)/36 (63%)	11 (52%)/10 (48%)		

IPAQ = International Physical Activity Questionnaire; * Denotes significant differences between the two groups. Data are expressed as means and standard deviations (SD).

**Table 2:** Median score of the single skills of the TGMD-3 for DSG and TDG.

Skill	DSG (*n* = 57)	TDG (*n* = 21)	Z-Value	*p*-Value	Cohen’s *d*
Running—RU *	4.0 (0.742)	8.0 (0.674)	−3.55	<0.001	0.878
Galloping—GA *	6.0 (0.579)	8.0 (0.719)	−3.40	<0.001	0.834
Hopping—HO *	3.0 (0.806)	6.0 (0.709)	−4.97	<0.001	1.362
Skipping—SK *	0.0 (0.362)	6.0 (0.414)	−5.14	<0.001	1.431
Horizontal Jumping—HJ *	4.0 (0.589)	6.0 (0.044)	−2.72	0.006	0.647
Sliding—SL *	6.0 (0.658)	6.0 (0.0)	−2.32	0.020	0.544
Two-hand striking—TH *	5.0 (0.771)	6.0 (0.291)	−2.03	0.041	0.472
One-hand striking—OH *	2.0 (0.505)	6.0 (0.354)	−5.63	<0.001	1.655
Stationary dribbling—SD *	3.0 (0.712)	6.0 (0.487)	−3.43	<0.001	0.843
Catching—CA *	3.0 (0.542)	4.0 (0.671)	−3.93	<0.001	0.994
Kicking—KI *	2.0 (0.452)	6.0 (0.500)	−4.49	<0.001	1.181
Overarm throwing—OT *	2.0 (0.417)	6.0 (0.704)	−5.63	<0.001	1.655
Underarm rolling—UR *	5.0 (0.570)	6.0 (0.940)	−4.48	<0.001	1.177

The data are medians and the standard error of the median (SEM). The significance level is 0.05. * Denotes significant differences between the two groups.

**Table 3:** Anthropometric characteristics of the DSG that completed the adapted karate training program.

	DS (*n* = 37)
Age [years]	26.2 (8.3)
Mass [kg]	67.0 (12.2)
Height [m]	1.56 (0.09)
BMI [kg/m^2^]	27.5 (5.4)
IPAQ	1699.4 (1519.6)
Females/Males	10 (27%)/27 (73%)

The data are means and standard deviations (SD).

**Table 4:** Single skills score before (DS-PRE) and after (DS-POST) the adapted karate training.

Skill	DSG-PRE (*n* = 37; 10F)	DSG-POST (*n* = 37; 10F)	Z-Value	*p*-Value	Cohen’s *d*
Running—RU *	5.5 (0.852)	7.5 (0.652)	−3.551	<0.001	0.906
Galloping—GA *	5.0 (0.788)	6.0 (0.682)	−1.536	0.125	0.363
Hopping—HO *	2.5 (0.848)	3.5 (0.797)	−1.804	0.071	0.429
Skipping—SK *	0.0 (0.371)	0.0 (0.585)	−0.960	0.337	0.225
Horizontal Jumping—HJ *	3.0 (0.787)	5.0 (0.574)	−3.153	<0.002	0.788
Sliding—SL *	6.0 (0.952)	8.0 (0.520)	−4.091	<0.001	1.081
Two-hand striking—TH *	4.0 (0.601)	5.0 (0.572)	−1.685	0.092	0.399
One-hand striking—OH *	3.0 (0.538)	5.0 (0.759)	−3.895	<0.001	1.016
Stationary dribbling—SD *	3.0 (0.639)	4.0 (0.506)	−2.332	0.019	0.563
Catching—CA *	2.0 (0.560)	4.0 (0.737)	−3.988	<0.001	1.046
Kicking—KI *	3.0 (0.560)	4.0 (0.481)	−3.283	<0.002	0.826
Overarm throwing—OT *	2.0 (0.442)	3.0 (0.770)	−1.788	0.074	0.425
Underarm rolling—UR *	5.0 (0.715)	6.0 (0.054)	−4.247	<0.001	1.135

The data are medians and the standard error of the median (SEM). * Denotes significant differences between pre and post intervention assessements. at the 0.05 level.

## Data Availability

The data presented in this study could be made available upon request to the corresponding author.
